# Trends in breast cancer screening during the COVID‐19 pandemic within a universally insured health system in the United States, 2017–2022

**DOI:** 10.1002/cam4.6487

**Published:** 2023-08-28

**Authors:** Vivitha Mani, Amanda Banaag, Satish Munigala, Ada Umoh, Andrew J. Schoenfeld, Christian L. Coles, Tracey Perez Koehlmoos

**Affiliations:** ^1^ Center for Health Services Research Uniformed Services University of the Health Sciences Bethesda Maryland USA; ^2^ The Henry M. Jackson Foundation for the Advancement of Military Medicine, Inc Bethesda Maryland USA; ^3^ Department of Orthopaedic Surgery Center for Surgery and Public Health Brigham and Women's Hospital, Harvard Medical School Boston Massachusetts USA

## Abstract

**Background:**

In the United States, breast cancer is the most commonly diagnosed cancer and second leading cause of cancer death in women. Early detection through mammogram screening is instrumental in reducing mortality and incidence of disease. The COVID‐19 pandemic posed unprecedented challenges to the provision of care, including delays in preventive screenings. We examined trends in breast cancer screening during the COVID‐19 pandemic in a universally insured national population and evaluated rates across racial groups and socioeconomic strata.

**Methods:**

In this retrospective open cohort study, we used the Military Health System Data Repository to identify female TRICARE beneficiaries ages 40–64 at average risk for breast cancer between FY2018 and FY2022, broken down into prepandemic (September 1, 2018–February 28, 2020), early pandemic (March 1, 2020–September 30, 2020), and late pandemic periods (October 1, 2020–September 30, 2022). The primary outcome was receipt of breast cancer screening.

**Results:**

Screening dropped 74% in the early pandemic period and 22% in the late pandemic period, compared with the prepandemic period. Compared with White women, Asian/Pacific Islander women were less likely to receive mammograms during the late pandemic period (0.92RR; 0.90–0.93 95%CI). American Indian/Alaska Native women remained less likely to receive screenings compared with White women during the early (0.87RR; 0.80–0.94 95% CI) and late pandemic (0.94RR, 0.91–0.98 95% CI). Black women had a higher likelihood of screenings during both the early pandemic (1.10RR; 1.08–1.12 95% CI) and late pandemic (1.12RR, 1.11–1.13 95% CI) periods compared with White women. During the early and late pandemic periods, disparities by rank persisted from prepandemic levels, with a decrease in likelihood of screenings across all sponsor ranks.

**Conclusion:**

Our results indicate the influence of race and socioeconomics on mammography screening during COVID‐19. Targeted outreach and further evaluation of factors underpinning lower utilization in these populations are necessary to improve access to preventative services across the population.

## 1 BACKGROUND

In the United States, breast cancer is the most commonly diagnosed cancer and second leading cause of cancer death in women.^1^, ^2^ It was predicted that, in 2022 alone, almost 290,000 new diagnoses and 43,000 deaths attributable to breast cancer would occur.^3^ Early detection through mammogram screening has been instrumental in reducing the incidence of advanced stage disease and mortality. Several medical organizations have published guidelines and recommendations on age and frequency of screening mammograms. The US Preventive Services Task Force, since 2016, has recommended biennial breast cancer screening for women aged 50–64 at average risk of disease, while the American Cancer Society recommends annual screening for women 45–54 years of age who are at average risk, with the option to switch to biennial mammograms at age 55.^4^, ^5^ There are, however, differences in screening rates across the country based on demographic factors.^6^, ^7^ Women in lower socioeconomic brackets are less likely to get screened for breast cancer, and differences in the quality of care received during and after screening visits have been reported for racial and ethnic minorities.^7^, ^8^

These pre‐existing challenges were likely further exacerbated by the COVID‐19 pandemic due to restrictions on in‐person evaluations and diversion of health care resources.^9^ This redirection of resources led to delays and cancelations of nonurgent procedures and services. Across the board, there was a significant decrease in preventive screenings and early diagnosis of chronic conditions.^9^, ^10^ Studies of claims data in April 2020 showed over 80% reduction in mammograms compared with February 2020.^11^ Much of this reduction was attributed to the onset of the pandemic, and it is estimated that this decrease alone could have resulted in 36,000 delayed breast cancer diagnoses.^11^ The challenges faced by the health care system during this period may have worsened existing disparities in access to health services, including many people who lost their health insurance as a result of job loss.^12^, ^13^

The Military Health System (MHS) is unique among other health systems in the United States in that it provides health care to a universally insured beneficiary population.^14^, ^15^ Through the TRICARE plan, beneficiaries can receive either direct care at military treatment facilities (MTFs) or in private civilian facilities where the plan serves as an insurance benefit. This system minimizes several barriers often encountered in civilian health care settings that prevent individuals from accessing and utilizing medical services, and also mitigate racial disparities in cancer screenings.^16^, ^17^ In this context, we sought to examine changes in breast cancer screening rates among TRICARE beneficiaries that occurred during the COVID‐19 pandemic. Given the nature of the MHS, studying the influence of the pandemic on care in the MHS may provide insight into the current state of preventive care across the nation. Based on previous work, we hypothesized that screening rates decreased during the pandemic, and that there were differences in screening uptake across different demographic categories.

## 2 METHODS

### 2.1 Study design, data source, and study population

This retrospective open cohort study analyzed administrative and health care claims data from the MHS Data Repository (MDR) for female TRICARE Prime beneficiaries ages 40–64 during fiscal years (FY) 2018–2022 (October 1, 2017–September 30, 2022). The MDR houses all health care encounter and claims data for MHS beneficiaries who received care at a military treatment facility (MTF) (direct care) or at a civilian fee‐for‐service treatment facility through their TRICARE benefit (private sector care (PSC)).^18^ Women ages 65 and older were excluded due to Medicare becoming the primary payer, resulting in a loss of transparency of care. Additionally, women who were beneficiaries of the National Guard or Reserves were excluded due to differences in access to care within the MHS.

**FIGURE 1:**
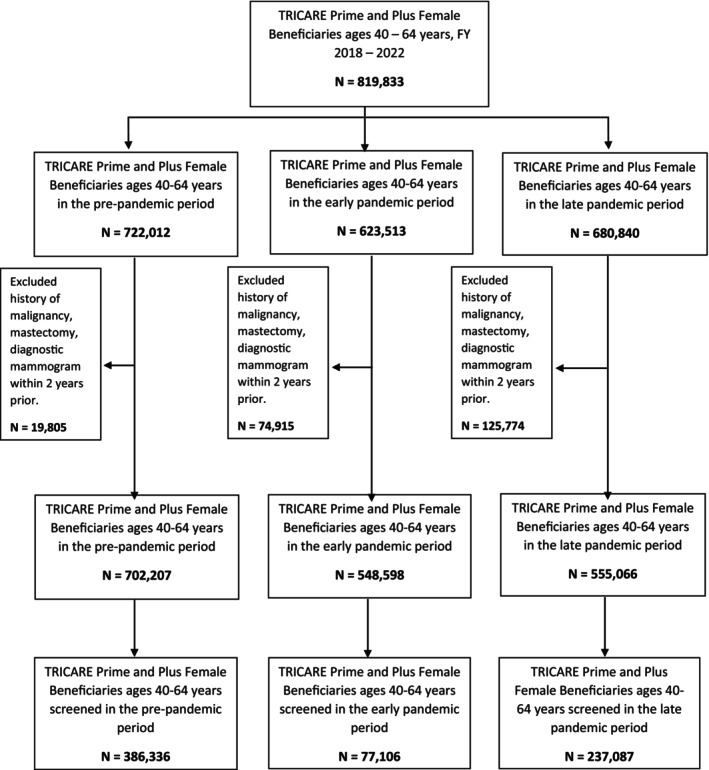
CONSORT diagram.

Utilizing Current Procedural Terminology/Healthcare Common Procedure Coding System (CPT/HCPCS) codes we identified all screening mammography claims for the study population between FY 2018 and 2022 (Table S1). Any women with a history of diagnostic mammography, history of malignancy, or mastectomy within 2 years prior to the screening period were excluded from the analysis (Figure 1; Table S1). Patient demographics such as age (categorized into 40–44, 45–49, 50–54, 55–59, and 60–64), race (White, Black, Asian/Pacific Islander, American Indian/Alaska Native, Other, and Unknown/Missing; self‐reported), beneficiary status (active duty, dependent, retiree, and other), sponsor rank as a proxy for socioeconomic status (Junior Enlisted, Senior Enlisted, Junior Officer, Senior Officer, Warrant Officer, and Other), and associated branch of service (Army, Navy, Marine Corps, Air Force) were obtained from the beneficiary's Defense Enrollment Eligibility Reporting System (DEERS) record during each FY and used in analysis. Data from the MDR does not specify ethnicity for the majority of beneficiaries, therefore we were unable to identify beneficiaries as Hispanic or non‐Hispanic. As such, White Hispanic women are grouped in the White cohort and Hispanic women of Afro‐Caribbean ancestry are categorized with Black women.

### 2.2 Study analyses

The primary outcome was receipt of screening mammography and the sociodemographic factors race and sponsor rank were considered the primary predictors. In our study race was missing for 42% of the study population. To resolve this issue, we utilized relational imputation by substituting patient's unknown or missing race with the known race of the sponsor, a method previously published in an analysis of mammography screening rates.^19^, ^20^ In this setting, and based on previously published work that supports use of sponsor rank as a proxy for socioeconomic status,^14^, ^15^, ^21^ enlisted personnel were considered proxies for lower socioeconomic strata. Study analyses included descriptive statistics on patient demographics and trend analysis of quarterly mammography screening rates overall and by race and rank. Binomial Poisson regressions were performed to calculate unadjusted and adjusted rate ratios and were used to assess for racial and socioeconomic disparities in screening mammography over the total study period and stratified by three summative periods, and the interactions between time periods and race and rank. Based on previously accepted cutoffs for the COVID‐19 pandemic,^22^ prepandemic (October 1, 2017–February 28, 2020), early pandemic (March 1, 2020–September 30, 2020), and late pandemic (October 1, 2020–September 30, 2022) were used to evaluate the effect of the COVID‐19 pandemic on screening mammography rates. Categorical age and beneficiary status were used as adjustment factors in the adjusted regression models. Additionally, adjusted regression models were used in sensitivity analyses of imputed race by comparing screening rates in those with a known race prior to imputation to those with an imputed race; these results can be found in Supplemental Materials (Table S2). Statistical significance was set at α < 0.05, and all analyses were performed using SAS, version 9.4. This study was reviewed and determined to be exempt by the Institutional Review Board at the Uniformed Services University of the Health Sciences.

## 3 RESULTS

We identified 819,833 women eligible for breast cancer screening during the study period (October 1, 2017–September 30, 2022): 702,207 in the prepandemic period, 548,598 during the early pandemic period, and 555,066 during the late pandemic period (Figure 1; Table 1). Among these, 386,336 (55.02%) were screened in the prepandemic period, 77,106 (14.06%) were screened in the early pandemic period, and 237,087 (42.71%) were screened in the late pandemic period; resulting in a ‐74% rate change in the early pandemic period and a ‐22% rate change in the late pandemic period compared to the proportion of women screened in the prepandemic period. The total study population consisted primarily of women who are ages 55–64, White, and in or associated with a Senior Enlisted rank (Table 1).

**TABLE 1:** Demographics of age eligible women for screening mammography from FY 2018 to 2022.

	Prepandemic period (*N* = 702,522)		Early pandemic period (*N* = 548,598)		Late pandemic period (*N* = 555,066)	
	*n* (col %) Not screened	*n* (col %) Screened	*n* (col %) Not screened	*n* (col %) Screened	*n* (col %) Not screened	*n* (col %) Screened
Total	315,871 (44.98)	386,336 (55.02)	471,492 (85.94)	77,106 (14.06)	317,979 (57.29)	237,087 (42.71)
Age group						
40–44	88,636 (28.06)	74,296 (19.23)	105,203 (22.31)	12,490 (16.20)	98,961 (31.12)	48,543 (20.47)
45–49	57,329 (18.15)	70,030 (18.13)	85,503 (18.13)	12,666 (16.43)	51,218 (16.11)	37,792 (15.94)
50–54	55,406 (17.54)	80,293 (20.78)	88,428 (18.75)	15,630 (20.27)	51,140 (16.08)	45,170 (19.05)
55–64	114,500 (36.25)	161,717 (41.86)	192,358 (40.80)	36,320 (47.10)	116,660 (36.69)	105,582 (44.53)
Patient's race						
White	97,390 (30.83)	131,269 (33.98)	156,599 (33.21)	25,578 (33.17)	101,506 (31.92)	76,961 (32.46)
Black	36,132 (11.44)	52,993 (13.72)	61,399 (13.02)	10,764 (13.96)	37,223 (11.71)	33,080 (13.95)
Asian/Pacific Islander	22,773 (7.21)	26,920 (6.97)	24,525 (5.20)	3908 (5.07)	17,428 (5.48)	11,114 (4.69)
American Indian/Alaska Native	1423 (0.45)	1466 (0.38)	2109 (0.45)	257 (0.33)	1579 (0.50)	965 (0.41)
Other	19,041 (6.03)	26,997 (6.99)	33,939 (7.20)	5087 (6.60)	22,399 (7.04)	15,716 (6.63)
Unknown/missing	146,680 (46.44)	153,477 (39.73)	192,921 (40.92)	31,512 (40.87)	137,844 (43.35)	99,251 (41.86)
Patient's race/sponsor's race (imputed race)						
White	199,383 (63.12)	239,603 (62.02)	292,188 (61.97)	47,927 (62.16)	197,628 (62.15)	147,658 (62.28)
Black	56,166 (17.78)	74,792 (19.36)	88,769 (18.83)	15,422 (20.00)	56,785 (17.86)	47,813 (20.17)
Asian/Pacific Islander	22,773 (7.21)	26,920 (6.97)	34,424 (7.30)	5249 (6.81)	25,845 (8.13)	15,657 (6.60)
American Indian/Alaska Native	2770 (0.88)	2596 (0.67)	3772 (0.80)	492 (0.64)	2898 (0.91)	1781 (0.75)
Other	24,426 (7.73)	32,582 (8.43)	41,513 (8.80)	6248 (8.10)	28,021 (8.81)	19,565 (8.25)
Unknown/Missing	10,353 (3.28)	9843 (2.55)	10,826 (2.30)	1768 (2.29)	6802 (2.14)	4613 (1.95)
Beneficiary status						
Active duty	10,268 (3.25)	15,364 (3.98)	17,457 (3.70)	2944 (3.82)	13,951 (4.39)	10,224 (4.31)
Dependent	272,053 (86.13)	333,621 (86.36)	405,691 (86.04)	67,142 (87.08)	270,009 (84.91)	205,993 (86.88)
Retiree	33,296 (10.54)	37,246 (9.64)	48,164 (10.22)	7005 (9.08)	33,778 (1062)	20,791 (8.77)
Other	254 (0.08)	105 (0.03)	180 (0.04)	1768 (2.29)	241 (0.08)	79 (0.03)
Rank group						
Junior Enlisted	6327 (2.00)	4582 (1.19)	7527 (1.60)	827 (1.07)	7021 (2.21)	3053 (1.29)
Senior Enlisted	233,515 (73.93)	269,929 (69.87)	341,805 (72.49)	53,523 (69.41)	235,393 (74.03)	164,777 (69.50)
Junior Officer	28,804 (9.12)	35,537 (9.20)	41,903 (8.89)	6816 (8.84)	29,023 (9.13)	21,658 (9.14)
Senior Officer	35,604 (11.27)	61,101 (15.82)	62,167 (13.19)	12,790 (16.59)	34,793 (10.94)	37,537 (15.83)
Warrant Officer	11,585 (3.67)	15,174 (3.93)	18,053 (3.83)	3148 (4.08)	11,725 (3.69)	10,050 (4.24)
Missing	36 (0.01)	13 (0.00)	<11	<11	24 (0.01)	12 (0.01)
Service						
Army	122,148 (38.67)	143,830 (37.23)	180,290 (38.24)	28,965 (37.57)	123,022 (38.69)	90,721 (38.26)
Air Force	91,907 (29.10)	120,393 (31.16)	140,238 (29.74)	22,853 (29.64)	94,283 (29.65)	67,859 (28.62)
Navy	73,285 (23.20)	85,591 (22.15)	106,660 (22.62)	17,781 (23.06)	71,408 (22.46)	54,276 (22.89)
Marine Corps	20,201 (6.40)	25,010 (6.47)	30,905 (6.55)	5117 (6.64)	20,828 (6.55)	15,944 (6.72)
Other	8330 (2.64)	11,512 (2.98)	13,399 (2.84)	2390 (3.10)	8438 (2.65)	8287 (3.50)

**FIGURE 2:**
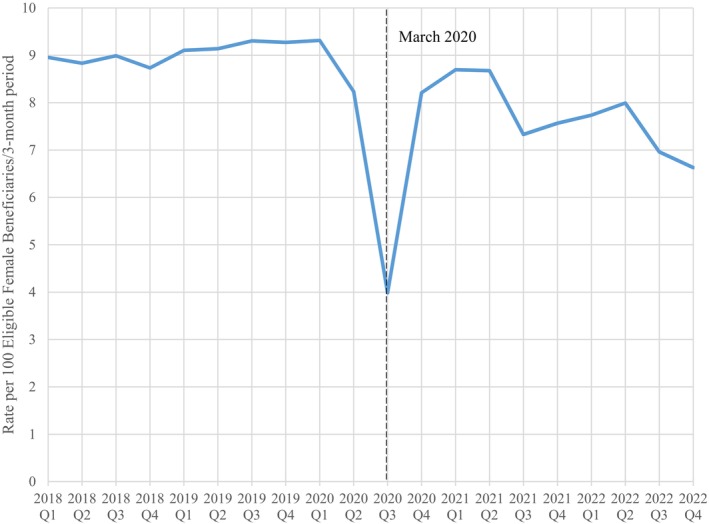
Total quarterly mammography screening rates in the MHS, FY 2018–2022.

**FIGURE 3:**
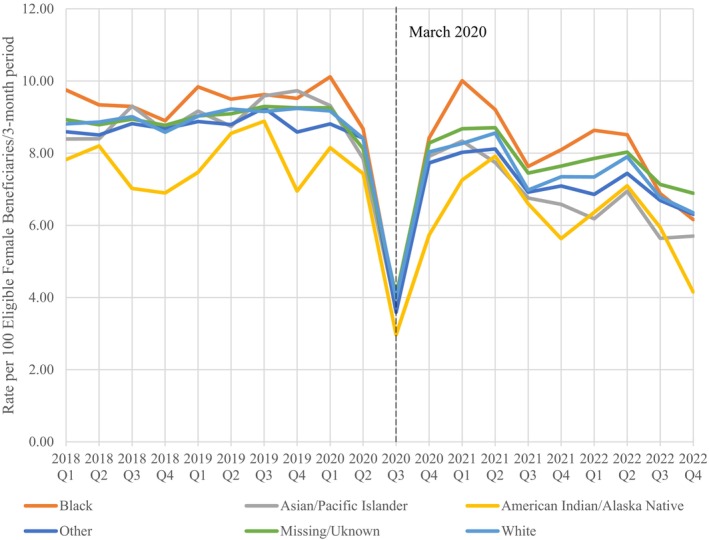
Quarterly mammography screening rates by Race, FY 2018–2022.

**FIGURE 4:**
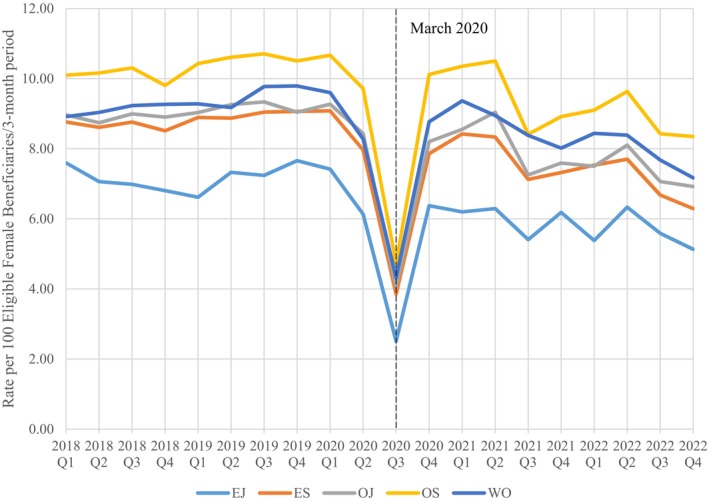
Quarterly mammography screening rates by Sponsor Rank, FY 2018–2022.

Figure 2 shows a graphical representation of the change in quarterly (per 3‐month period) mammography screening rates across the study period. Prior to COVID‐19 pandemic, the screening rate for breast cancer was approximately 9 per 100 eligible women. At the onset of the pandemic in March 2020, the rate noticeably drops down to about four per 100 eligible women, after which rates begin to rise again through September 30, 2020. This pattern is also seen across the study period when broken down by race (Figure 3) and the patient's rank or sponsor's rank (Figure 4).

Table 2 displays the unadjusted and adjusted rate ratio (RR) results of race, SES, and time period as predictors for mammography screenings over the full study period. Adjusted analyses of screening rates showed that, compared to White women, Black women (1.09 RR; 1.09–1.10 95% CI) and women of ‘other’ race (1.02 RR; 1.02–1.03 95% CI) were more likely to receive mammography screenings, while Asian/Pacific Islander (0.98 RR; 0.97–0.98 95% CI) and American Indian/Alaskan Native (0.92 RR; 0.90–0.95 95% CI) women were less likely to receive mammography screenings (Table 2). In the evaluation of SES on mammography screening rates, adjusted analyses showed all ranks below Senior Officer were less likely to receive mammography screenings over the full study period (Table 2). In the evaluation of the effects of the COVID‐19 pandemic, mammography screenings rates were significantly less likely during both the early (0.25 RR, 0.24–0.25 95% CI) and late pandemic (0.78 RR, 0.78–0.78 95% CI) periods compared to the prepandemic period (Table 2). In the evaluation of the interaction between time and race and rank, both interactions were statistically significant in the regression models (type 3 chi‐square *p*‐values <0.05); and, significant differences in rates were observed across all races and ranks in both the early and late pandemic periods compared to White and Senior Officer women in the prepandemic period (chi‐square *p*‐values <0.05).

**TABLE 2:** Breast cancer screening rate ratio results for MHS beneficiaries ages 40–64, FY 2018–2022.

	Total study period, unadjusted			Total study period, adjusted		
	RR	95% CI		RR	95% CI	
Race/sponsor's race (imputed race)						
White (ref)	1.00	1.00	1.00	1.00	1.00	1.00
Black	1.05*	1.05	1.06	1.09*	1.09	1.10
Asian/Pacific Islander	0.95*	0.94	0.96	0.98*	0.97	0.98
American Indian/Alaska Native	0.89*	0.86	0.91	0.92*	0.90	0.95
Other	0.99	0.99	1.00	1.02*	1.02	1.03
Rank						
Junior Enlisted	0.61*	0.60	0.63	0.66*	0.65	0.68
Senior Enlisted	0.83*	0.83	0.83	0.83*	0.83	0.84
Junior Officer	0.87*	0.86	0.87	0.89*	0.89	0.90
Senior Officer (ref)	1.00	1.00	1.00	1.00	1.00	1.00
Warrant Officer	0.90*	0.89	0.91	0.91*	0.91	0.92
Time period						
Prepandemic (ref)	1.00	1.00	1.00	1.00	1.00	1.00
Early pandemic	0.25*	0.25	0.25	0.25*	0.24	0.25
Late pandemic	0.78*	0.77	0.78	0.78*	0.78	0.78

*Note*: Multivariate binomial Poisson regression models were adjusted by categorical age and beneficiary status.

Table 3 displays the stratified adjusted analyses evaluating patient demographic associations of screening rates within each time period. Adjusted analyses of screening rates showed that, prior to the onset of the COVID‐19 pandemic, Black (1.07 RR; 1.07–1.08 95% CI), Asian/Pacific Islander (1.02 RR; 1.01–1.03 95% CI), and ‘other’ race (1.06 RR, 1.06–1.07 95% CI) women were more likely to receive breast cancer screening compared to White women (Table 3). In contrast, American Indian/Alaska Native women were less likely to get screened (0.91 RR; 0.89–0.94 95% CI). Compared to White women, Black women had a higher likelihood of mammography screenings during both the early pandemic (1.10 RR; 1.08–1.12 95% CI) and late pandemic (1.12 RR, 1.11–1.13 95% CI) periods; representing a 3% and 5% increase in likelihood from the prepandemic period, respectively. Asian/Pacific Islander women did not experience a significant difference in screenings during the early pandemic period, however they were less likely to receive mammography screenings during the late pandemic period (0.92 RR; 95% CI 0.90–0.93 95% CI) compared to White women; representing a 10% decrease in likelihood from the prepandemic period. American Indian/Alaska Native women remained less likely to receive screenings compared to White women during the early pandemic (0.87 RR; 0.80–0.94 95% CI) and the late pandemic (0.94 RR, 0.91–0.98 95% CI) periods; a 4% decrease and 3% increase in likelihood, respectively, from the prepandemic period. During all periods all sponsor ranks (Junior Enlisted, Senior Enlisted, Junior Officer, and Warrant Officer) had lower rates of screening compared to Senior Officers (Table 3). During both the early and late pandemic periods, a decrease in the likelihood of screenings was observed across all ranks from the prepandemic period (Table 3).

**TABLE 3:** Pandemic period stratified breast cancer screening rate ratio results for MHS beneficiaries ages 40–64, FY 2018–2022.

	Prepandemic period, adjusted			Early pandemic period, adjusted			Late pandemic period, adjusted		
	RR	95% CI		RR	95% CI		RR	95% CI	
Race/sponsor's race (imputed race)									
White (ref)	1.00	1.00	1.00	1.00	1.00	1.00	1.00	1.00	1.00
Black	1.07*	1.07	1.08	1.10*	1.08	1.12	1.12*	1.11	1.13
Asian/Pacific Islander	1.02*	1.01	1.03	0.97	0.95	1.00	0.92*	0.90	0.93
American Indian/Alaska Native	0.91*	0.89	0.94	0.87*	0.80	0.94	0.94*	0.91	0.98
Other	1.06*	1.06	1.07	0.96*	0.93	0.98	0.98*	0.97	0.99
Rank									
Junior Enlisted	0.70*	0.68	0.72	0.60*	0.56	0.65	0.63*	0.61	0.65
Senior Enlisted	0.86*	0.85	0.86	0.80*	0.78	0.81	0.81*	0.80	0.81
Junior Officer	0.91*	0.90	0.91	0.86*	0.84	0.89	0.88*	0.87	0.89
Senior Officer (ref)	1.00	1.00	1.00	1.00	1.00	1.00	1.00	1.00	1.00
Warrant Officer	0.91*	0.90	0.92	0.89*	0.86	0.93	0.92*	0.90	0.93

*Note*: Pre‐, early, and late pandemic period multivariate binomial Poisson regression models were adjusted by categorical age and beneficiary status.

## 4 DISCUSSION

Our analysis of TRICARE beneficiary data showed an overall reduction in breast cancer screening rates in both the early pandemic and late pandemic periods when compared to prepandemic rates. The sharp decline in screening around March and April 2020 coincides with the onset of COVID‐19 restrictions and is consistent with previous investigations regarding the impact of the pandemic on cancer screening rates. Although screening rates appear to trend upward immediately after the initial decline, our findings indicate worrisome and persistent reductions for several specific subgroups in the US demographic.

For example, Asian/Pacific Islanders experienced a significant decrease in screening during the pandemic period. While screening rates among American Indian/Alaskan Native women increased slightly in the late pandemic period compared to prepandemic levels, overall they were still less likely to get screened both prior to and during the pandemic. Disparities by sponsor rank, used as a proxy for socioeconomic status, persisted between prepandemic and early and late pandemic periods, with women from lower socioeconomic strata less likely to be screened across all time periods. Although screening rates are trending back upward after the initial onset of COVID‐19, important disparities persist for Asian/Pacific Islanders, American Indian/Alaskan Natives, and those in lower socioeconomic strata.

Our findings are consistent with previous studies that assessed the impact of the pandemic on breast cancer screening services. For example, a study of data from the National Breast and Cervical Cancer Early Detection Program showed a sharp decrease in screenings in March–April 2020 when compared to the 5‐year average prior to COVID‐19, dropping 87% in April.^13^ Similarly, a study by the National Cancer Institute's Population‐based Research to Optimize the Screening Process consortium found a 96% decrease in screening in April and May 2020 compared to rates in 2019.^23^ These results, combined with our determination of a significant decline in screening among Asian/Pacific Islander women may indicate that Asian women experienced the largest initial decrease in screening as well as the slowest rebound in subsequent years.^13^, ^24^, ^25^, ^26^ Unlike other racial minority groups in the United States, low screening rates among Asian/Pacific Islanders cannot be fully explained by the usual barriers such as income and health care access, and that cultural and social factors may be playing more of a role, particularly during the early days of COVID‐19.^27^ At the start of the pandemic there was a surge in anti‐Asian violence, and some literature cites this rise in racial violence and fear among the Asian American population as an additional barrier in deciding to seek preventive services and other medical care.^27^, ^28^

While our findings show that American Indian/Alaskan Natives continued to be screened less frequently compared to other groups both prior to the pandemic and during both early and late pandemic, we saw a slight increase in screening uptake during the pandemic. Other literature on the topic has generally shown a substantial decrease in screening mammograms among this population.^13^, ^26^, ^29^ One study comparing screening rates among a cohort of women in Washington state, for example, found a 60% reduction in screening mammograms among American Indian/Alaska Native women.^30^ These studies cite the primary reason for this as American Indian/Alaskan Natives being less likely to have health insurance or adequate access to health care services compared to other racial groups in the United States.^25^, ^30^, ^31^, ^32^ Our study population may not show the same large decline during the COVID‐19 period due to the nature of universal insurance in the setting of TRICARE and associated reductions in barriers to accessing care. However, the low rates of screening uptake even in this setting is indicative of other factors that likely contribute to differences in the use of screening mammograms in this minority group.

It is important to note that our results show that the breast cancers screening rate for Black women rose during the pandemic and exceeded those for White women both prior to and during the pandemic. This is in contrast to data from previously published studies which indicated that Black women are less likely to be screened for breast cancer than White women.^33^, ^34^ One possible explanation for the higher screening mammography rates seen in our study population may have to do with insurance coverage. In the United States, insurance coverage among Black people and other racial minorities is much lower compared with White people, posing a significant barrier to medical care and preventive services. Several studies, including one conducted by Agrawal et al in Texas, have demonstrated positive associations between health insurance coverage and adherence to breast cancer screening among Black women.^35^, ^36^ Therefore, it would stand to reason that Black TRICARE beneficiaries would be more likely to seek out and receive screening services, since the barrier of insurance coverage is eliminated in the MHS.

While we recognize further studies are needed to fully validate these findings, the disparities in mammography screenings during the COVID‐19 period in specific racial and socioeconomic groups identified here represent best available evidence at this time. Particularly among Asian/Pacific Islanders, American Indian/Alaskan Natives and those of lower socioeconomic strata, the need for both targeted outreach as well as further evaluation of the drivers of low screening uptake during COVID‐19 may prove beneficial for improving access to preventative services in these populations.

We recognize several limitations to this study. Given the study design and a reliance on health care claims data, we were restricted to the information that was available in the MHS Data Repository without being able to collect additional information in the event of missing data, and could not account for changing trends in screening that may have been occurring prior to the study period. Additionally, the time range used for the pandemic period was restricted to March 2020–September 2022 as this was the most recent data available, so we were unable to evaluate trends in screening rates beyond this time window. Due to the nature of data reporting in the MDR, ethnicity of beneficiaries was unavailable for study analysis and therefore we were unable to parse out screening rates for Hispanic patients separate from non‐Hispanic White and non‐Hispanic Black women. Our study population was also limited to women who had been screened via mammography only. However, it is unlikely that this would have a significant impact on our determinations given that mammograms are the most common modality for breast cancer screening. We also restricted analysis to women at average risk of breast cancer diagnosis since the inclusion of individuals at higher risk would have likely artificially increased screening rates and impaired generalizability. It is possible that since we only used a 2‐year period prior to screening to assess for exclusionary criteria, some people with a recorded history of malignancy or mastectomy prior to that 2‐year period may have been included in our dataset; however, we do not anticipate this to have a significant impact on the findings. And lastly, we recognize the utilization of relational imputation for patient's missing race is imperfect and does not account for interracial couples or marriages.

## 5 CONCLUSION

We found significant reductions in breast cancer screening within the MHS in the time period 2020–2022, which coincides with the onset of the COVID‐19 pandemic. We additionally identified new and persistent racial and socioeconomic disparities in the uptake of breast cancer screening among Asians/Pacific Islanders, American Indians/Alaskan Natives, and individuals from low socioeconomic background. Targeted outreach, as well as further evaluation of the factors underpinning lower utilization in these populations are necessary to improve access to preventative services within the MHS and nationwide.

## DISCLAIMER

The contents, views, or opinions expressed in this manuscript are those of the author(s) and do not necessarily reflect official policy or position of Uniformed Services University of the Health Sciences, the Department of Defense, or Departments of the Army, Navy, or Air Force, or the Henry M. Jackson Foundation for the Advancement of Military Medicine, Inc. Mention of trade names, commercial products, or organizations does not imply endorsement by the U.S. Government.

## AUTHOR CONTRIBUTIONS

**Vivitha Mani:** Conceptualization (equal); writing – original draft (equal); writing – review and editing (equal). **Amanda Banaag:** Conceptualization (equal); data curation (lead); formal analysis (lead); methodology (lead); writing – original draft (equal); writing – review and editing (equal). **Satish Munigala:** Conceptualization (equal); writing – review and editing (equal). **Ada Umoh:** Conceptualization (equal); methodology (supporting); writing – review and editing (equal). **Andrew J Schoenfeld:** Writing – review and editing (equal). **Christian L Coles:** Conceptualization (equal); methodology (supporting); writing – review and editing (equal). **Tracey Perez Koehlmoos:** Conceptualization (equal); methodology (supporting); resources (lead); supervision (lead); writing – review and editing (equal).

## FUNDING INFORMATION

This work was funded by the Department of Defense, Defense Health Agency (award #HU0012120089).

## CONFLICT OF INTEREST STATEMENT

The authors declare they have no conflicts of interest.

## Supporting information


Tables S1–S2

## Data Availability

Data sharing is not applicable to this article as no new data were created or analyzed in this study.

## References

[1] Giaquinto AN , Sung H , Miller KD , et al. Breast cancer statistics, 2022. CA Cancer J Clin. 2022;72(6):524‐541. doi:10.3322/caac.21754 36190501

[2] Sung H , Ferlay J , Siegel RL , et al. Global cancer statistics 2020: GLOBOCAN estimates of incidence and mortality worldwide for 36 cancers in 185 countries. CA Cancer J Clin. 2021;71(3):209‐249. doi:10.3322/caac.21660 33538338

[3] Siegel RL , Miller KD , Fuchs HE , Jemal A . Cancer statistics, 2022. CA Cancer J Clin. 2022;72(1):7‐33. doi:10.3322/caac.21708 35020204

[4] Siu AL . Force USPST. Screening for breast cancer: U.S. preventive services task force recommendation statement. Ann Intern Med. 2016;164(4):279‐296. doi:10.7326/M15-2886 26757170

[5] Society AC . ACS Breast Cancer Screening Guidelines. 2022 Accessed December 13, 2022. https://www.cancer.org/cancer/breast‐cancer/screening‐tests‐and‐early‐detection/american‐cancer‐society‐recommendations‐for‐the‐early‐detection‐of‐breast‐cancer.html

[6] Ma ZQ , Richardson LC . Cancer screening prevalence and associated factors among US adults. Prev Chronic Dis. 2022;19:E22. doi:10.5888/pcd19.220063 35446757 PMC9044902

[7] Ramachandran A , Snyder FR , Katz ML , et al. Barriers to health care contribute to delays in follow‐up among women with abnormal cancer screening: data from the patient navigation research program. Cancer. 2015;121(22):4016‐4024. doi:10.1002/cncr.29607 26385420 PMC4675460

[8] Grabinski VF , Brawley OW . Disparities in breast cancer. Obstet Gynecol Clin N Am. 2022;49(1):149‐165. doi:10.1016/j.ogc.2021.11.010 35168767

[9] Barsouk A , Saginala K , Aluru JS , Rawla P , Barsouk A . US cancer screening recommendations: developments and the impact of COVID‐19. Med Sci (Basel). 2022;10(1):16. doi:10.3390/medsci10010016 35323215 PMC8949858

[10] Peng SM , Yang KC , Chan WP , et al. Impact of the COVID‐19 pandemic on a population‐based breast cancer screening program. Cancer. 2020;126(24):5202‐5205. doi:10.1002/cncr.33180 32914864

[11] Aitken M , Kleinrock M . Shifts in healthcare demand, delivery and care during the COVID‐19 era. 2020.

[12] Cancino RS , Su Z , Mesa R , Tomlinson GE , Wang J . The impact of COVID‐19 on cancer screening: challenges and opportunities. JMIR Cancer. 2020;6(2):e21697. doi:10.2196/21697 33027039 PMC7599065

[13] DeGroff A , Miller J , Sharma K , et al. COVID‐19 impact on screening test volume through the National Breast and cervical cancer early detection program, January‐June 2020, in the United States. Prev Med. 2021;151:106559. doi:10.1016/j.ypmed.2021.106559 34217410 PMC9026719

[14] Chaudhary MA , de Jager E , Bhulani N , et al. No racial disparities In surgical care quality observed after coronary artery bypass grafting In TRICARE patients. Health Aff (Millwood). 2019;38(8):1307‐1312. doi:10.1377/hlthaff.2019.00265 31381404

[15] Schoenfeld AJ , Jiang W , Harris MB , et al. Association between race and postoperative outcomes in a universally insured population versus patients in the state of California. Ann Surg. 2017;266(2):267‐273. doi:10.1097/sla.0000000000001958 27501169

[16] Adirim T . A military health system for the twenty‐first century. Health Aff (Millwood). 2019;38(8):1268‐1273. doi:10.1377/hlthaff.2019.00302 31381414

[17] Tanielian T , Farmer C . The US military health system: promoting readiness and providing health care. Health Aff (Millwood). 2019;38(8):1259‐1267. doi:10.1377/hlthaff.2019.00239 31381396

[18] MDR Fact Sheet . Updated March 2019. Accessed March 13, 2023. https://www.health.mil/Reference‐Center/Fact‐Sheets/2019/03/27/MDR

[19] Bytnar JA , Byrne C , Olsen C , et al. The impact of mammography screening guideline changes in a universally insured population. J Womens Health (Larchmt). 2021;30(12):1720‐1728. doi:10.1089/jwh.2020.8546 33600239 PMC9839342

[20] Huang F , Kim JS . Differences in prevalence, treatment, and outcomes of asthma among a diverse population of children with equal access to care: findings from a study in the military health system. Pediatrics. 2011;128:S125‐S126. doi:10.1542/peds.2011-2107EEE 20530290

[21] Schoenfeld AJ , Kaji AH , Haider AH . Practical guide to surgical data sets: military health system Tricare encounter data. JAMA Surg. 2018;153(7):679‐680. doi:10.1001/jamasurg.2018.0480 29617526

[22] Crawford AM , Lightsey Iv HM , Xiong GX , et al. Changes in elective and urgent surgery among TRICARE beneficiaries during the COVID‐19 pandemic. Mil Med. 2022;188:e2397‐e2404. doi:10.1093/milmed/usac391

[23] Corley DA , Sedki M , Ritzwoller DP , et al. Cancer screening during the coronavirus Disease‐2019 pandemic: a perspective from the National Cancer Institute's PROSPR consortium. Gastroenterology. 2021;160(4):999‐1002. doi:10.1053/j.gastro.2020.10.030 33096099 PMC7575503

[24] Grimm LJ , Lee C , Rosenberg RD , et al. Impact of the COVID‐19 pandemic on breast imaging: an analysis of the National Mammography Database. J Am Coll Radiol. 2022;19(8):919‐934. doi:10.1016/j.jacr.2022.04.008 35690079 PMC9174535

[25] Sprague BL , Lowry KP , Miglioretti DL , et al. Changes in mammography use by Women's characteristics during the first 5 months of the COVID‐19 pandemic. J Natl Cancer Inst. 2021;113(9):1161‐1167. doi:10.1093/jnci/djab045 33778894 PMC8083761

[26] Lacson R , Shi J , Kapoor N , Eappen S , Boland GW , Khorasani R . Exacerbation of inequities in use of diagnostic radiology during the early stages of reopening after COVID‐19. J Am Coll Radiol. 2021;18(5):696‐703. doi:10.1016/j.jacr.2020.12.009 33482115 PMC7834847

[27] Lee RJ , Madan RA , Kim J , Posadas EM , Yu EY . Disparities in cancer care and the Asian American population. Oncologist. 2021;26(6):453‐460. doi:10.1002/onco.13748 33683795 PMC8176990

[28] Saw A , Yi SS , Ðoàn LN , et al. Improving Asian American health during the Syndemic of COVID‐19 and racism. eClinicalMedicine. 2022;45:101313. doi:10.1016/j.eclinm.2022.101313 35233516 PMC8881903

[29] Star J , Bandi P , Siegel RL , et al. Cancer screening in the United States during the second year of the COVID‐19 pandemic. J Clin Oncol. 2023;JCO2202170. doi:10.1200/JCO.22.02170 PMC10911528 36821800

[30] Amram O , Robison J , Amiri S , Pflugeisen B , Roll J , Monsivais P . Socioeconomic and racial inequities in breast cancer screening during the COVID‐19 pandemic in Washington state. JAMA Netw Open. 2021;4(5):e2110946. doi:10.1001/jamanetworkopen.2021.10946 34028552 PMC8144923

[31] Patt D , Gordan L , Patel K , et al. Considerations to increase rates of breast cancer screening across populations. Am J Manag Care. 2022;28(3):Sp136‐sp138. doi:10.37765/ajmc.2022.88855 35285592

[32] Schifferdecker KE , Vaclavik D , Wernli KJ , et al. Women's considerations and experiences for breast cancer screening and surveillance during the COVID‐19 pandemic in the United States: a focus group study. Prev Med. 2021;151:106542. doi:10.1016/j.ypmed.2021.106542 34217409 PMC8721569

[33] Miller BC , Bowers JM , Payne JB , Moyer A . Barriers to mammography screening among racial and ethnic minority women. Soc Sci Med. 2019;239:112494. doi:10.1016/j.socscimed.2019.112494 31513931

[34] Tangka FK , Subramanian S , Mobley LR , et al. Racial and ethnic disparities among state Medicaid programs for breast cancer screening. Prev Med. 2017;102:59‐64. doi:10.1016/j.ypmed.2017.06.024 28647544 PMC5840870

[35] Agrawal P , Chen TA , McNeill LH , et al. Factors associated with breast cancer screening adherence among church‐going African American women. Int J Environ Res Public Health. 2021;18(16):8494. doi:10.3390/ijerph18168494 34444241 PMC8392666

[36] Ko NY , Hong S , Winn RA , Calip GS . Association of Insurance Status and Racial Disparities with the detection of early‐stage breast cancer. JAMA Oncol. 2020;6(3):385‐392. doi:10.1001/jamaoncol.2019.5672 31917398 PMC6990828

