# Supplementary material for: Trends in breast cancer screening during the COVID‐19 pandemic within a universally insured health system in the United States, 2017–2022
Source: Cancer Med. 2023 Aug 28;12(18):19126–36. doi: 10.1002/cam4.6487 (PMC10557872; doi:10.1002/cam4.6487)
Supplement: Supplementary file 1 — Tables S1–S2 [file CAM4-12-19126-s001.docx]

**Supplemental Table 1. ICD-10, CPT/HCPCS, and MS-DRG Codes Used for Inclusion and Exclusion Criteria**

| **Code Description** | **Codes** |
| --- | --- |
| **ICD-10 Codes** | |
| Encounter for screening for malignant neoplasms of breast | Z12.3, Z12.31, Z12.39 |
| Localized swelling, mass and lump, trunk | R22.2 |
| Acquired absence of breast and nipple | Z90.11 – Z90.13 |
| **CPT/HCPCS Codes** | |
| Screening Mammography | 77063, 77067, G0202 |
| Diagnostic Mammography | 77061, 77062, 77065, 77066, G0204, G0206 |
| Mastectomy | 19303 – 19307 |
| **MS-DRG Codes** | |
| Mastectomy | 582, 583 |

**Supplemental Table 2. Sensitivity Analysis: Breast Cancer Screening Rate Ratio Results for MHS Beneficiaries Ages 40 to 64, FY 2018-2022**

|  | **Patient’s with a Known Race, Adjusted** | | | **Patient’s with a Missing Race, Adjusted** | | |
| --- | --- | --- | --- | --- | --- | --- |
| **Race/Sponsor's Race** | **RR** | **95% CI** | | **RR** | **95% CI** | |
| White (ref) | 1.00 | 1.00 | 1.00 | 1.00 | 1.00 | 1.00 |
| Black | 1.10* | 1.09 | 1.11 | 1.04* | 1.03 | 1.05 |
| Asian/Pacific Islander | 0.98* | 0.96 | 0.98 | 0.93* | 0.92 | 0.95 |
| American Indian/Alaska Native | 0.92* | 0.89 | 0.95 | 0.93* | 0.90 | 0.97 |
| Other | 0.99 | 0.99 | 1.00 | 1.00 | 0.98 | 1.02 |
| **Rank** |  |  |  |  |  |  |
| Junior Enlisted | 0.64* | 0.63 | 0.66 | 0.75* | 0.73 | 0.77 |
| Senior Enlisted | 0.84* | 0.84 | 0.85 | 0.83* | 0.82 | 0.83 |
| Junior Officer | 0.90* | 0.89 | 0.90 | 0.89* | 0.88 | 0.90 |
| Senior Officer (ref) | 1.00 | 1.00 | 1.00 | 1.00 | 1.00 | 1.00 |
| Warrant Officer | 0.91* | 0.90 | 0.93 | 0.91* | 0.89 | 0.93 |
| Time Period |  |  |  |  |  |  |
| Pre-COVID-19 (ref) | 1.00 | 1.00 | 1.00 | 1.00 | 1.00 | 1.00 |
| Early COVID-19 | 0.24* | 0.23 | 0.24 | 0.27* | 0.26 | 0.27 |
| Late COVID-19 | 075* | 0.74 | 0.75 | 0.84* | 0.83 | 0.84 |

Note: Multivariate Poisson regression models were adjusted by categorical age, and beneficiary status.
